# The T-loop in details

**DOI:** 10.1590/2177-6709.23.1.108-117.sar

**Published:** 2018

**Authors:** Amanda Frizzo Viecilli, Maria Perpétua Mota Freitas

**Affiliations:** 1 Private practice (Canoas/RS, Brazil).; 2 Universidade Luterana do Brasil, Dentistry Course (Canoas/RS, Brasil)

**Keywords:** Orthodontic anchoring procedures, Orthodontic space closure, Orthodontic appliance design, Tooth movement

## Abstract

**Introduction:**

The T-loop as designed by Burstone is a space closure spring used in the rational application of orthodontic biomechanics. Despite the diversity of studies, there is still no consensus on the optimal parametric characteristics for its conformation.

**Objective:**

This study aimed at reviewing the literature on the force systems released by different conformations of the T-loop, according to the type of anchorage and the main characteristics and factors that influence them.

**Results:**

Comparing the studies, the need for standardization was perceived in the methodology to shape the loops, regarding the variables that influence the force system. Most of the experimental studies with this loop do not report the vertical movement, nor the steps and angles that occur in the brackets. Clinical studies have obtained more variable results in relation to vertical acting forces, considering the influence of chewing.

**Conclusion:**

There is great potential for future studies with this type of loop, especially using nickel-titanium alloys, in order to achieve a pure translational movement without friction, with optimal and constant levels of force.

## RESUMO

**Introdução:**

a alça T preconizada por Burstone é um instrumento de fechamento de espaços utilizado na aplicação racional da biomecânica ortodôntica, dentro da técnica do arco segmentado. Apesar da diversidade de estudos, ainda não há consenso quanto às características paramétricas ótimas para a sua conformação.

**Objetivo:**

esse estudo teve como objetivo revisar a literatura sobre os sistemas de forças liberados por diferentes conformações da alça T, de acordo com o tipo de ancoragem necessária, salientando suas principais características e os fatores que as influenciam.

**Resultados:**

comparando-se os estudos, foi perceptível a necessidade de padronização da metodologia usada para conformar as alças, levando em consideração as variáveis que exercem influência no sistema de forças, de forma a se obter sistemas mais acurados, permitindo a realização de testes mais precisos e uma comparação mais eficaz dos estudos. A maioria dos estudos experimentais com esse tipo de alça não relata a movimentação vertical, nem os degraus e ângulos que ocorrem nos braquetes. Estudos clínicos obtiveram resultados menos rígidos em relação às forças verticais atuantes, levando em consideração a influência da mastigação.

**Conclusões:**

há grande potencial para futuros estudos com esse tipo de alça, especialmente utilizando ligas de níquel-titânio, para que se chegue cada vez mais perto de um movimento de translação puro e sem atrito, com níveis de força ótimos e constantes.

## INTRODUCTION

Orthodontic movement is defined by the effect of the force system on the tooth and the consequent responses of the adjacent structures.^1^ For that reason, effective space closure is challenging, and can be optimized when there is control and predictability of the force system.^2^ The released forces must be continuous and the center of rotation of the tooth must be constant to release biologically favorable forces that does not continually modify the stress areas of the periodontal ligament.^3^

The simplest way to determine and visualize the force system is utilizing two groups of teeth, to obtain one center of resistance and one center of rotation in each unit. This is possible using the segmented arch approach. Moreover, the greater interbrackets distance and smaller load/deflection rates of the loops are favorable to the dental movement biology.^4^ Pre-calibrated loops, as the T-loop, are an important part of this technical approach.^5^

Different T-loops designs have been studied in the literature regarding their parametric characteristics. However, there is still no consensus on which height, apical length, preactivation, material and cross-section are more adequate. In their studies, several authors did not evaluate some characteristics that directly influence the appliance force system, such as the neutral position and the possibility of permanent deformation. In addition, an important attribute of the T-loop is the possibility of obtaining, with different preactivations or with the eccentric positioning of the spring, differential moments or differential forces,^2^ to achieve a differential space closure, that is, a space closure greater in one unit compared to the other.^6^ Thus, the aim of this study was to review the literature on the force systems obtained in different studies related to the segmented T-loop, specially regarding the main factors that influence it. 

## MOMENT-TO-FORCE RATIO AND IDEAL FORCE MAGNITUDE

There are issues regarding loops that should be considered, which directly influence the dental movement obtained with their activation. When choosing the ideal space closure method, the main variable that must be considered is the distance between the line of action of the equivalent resultant force and the orthodontic bracket, known as the moment-to-force ratio (M/F).^6^ In general, in cases where a controlled inclination is desired, and the distance between the bracket and the center of resistance of the tooth is 10mm, a M/F of 7mm is indicated, and, for translation, a M/F of 10mm.^7^

There is still no consensus in the literature on the magnitude of the loads that must be applied for space closure^3^. A systematic review did not find enough data to determine the magnitude of the force.^8^ This was probably due to problems in the concepts of force, load and stress used in Orthodontics.^9^ Apparently, inclination movement requires less loads than the translation movement. This is compatible with the results of Viecilli et al.^10^

## T-LOOP PARAMETRIC CHARACTERISTICS

T-loops were developed by applying engineering principles to increase M/F ratios and optimize their design. For example, the vertical height of the loop directly influences the M/F ratio. As the height increases, a greater M/F ratio is obtained.^5^ ^,^ ^11^ ^,^ ^12^ This occurs because the wire becomes more flexible and releases less force^11^ ^,^ ^13^. Another advantage of increasing the height of the loop is that it decreases the probability of activation without reaching the plastic deformation. The mean heights varied, according to the studies, between 6 and 10.45mm.^5^ ^,^ ^11^ ^,^ ^13^ ^-^ ^20^ ^,^ ^22^ ^-^ ^30^

The M/F ratio increases when adding apical length, but never reaches the absolute value of the height. Consequently, within the anatomical limits, even increasing the horizontal length and the height of the loop is not enough to produce ideal M/F for controlled inclination and translation. Because of this, preactivation bends were suggested.^5^ In the analyzed studies, apical length ranged from 10 to 16 mm.^5^ ^,^ ^11^ ^,^ ^13^ ^-^ ^20^ ^,^ ^22^ ^-^ ^30^

The horizontal length of the loop is determined by the bracket distance and the teeth positioning. The M/F ratio tends to decrease as the interbrackets distance increases, but with less influence than the height and apical length. However, it is convenient to have a larger interbrackets distance because it dramatically reduces the load/deflection rate, releasing a more constant magnitude of force.^5^ ^,^ ^11^ It is recommended to use stiffer wires in the horizontal arms and lighter wires in the loop region.^6^ In the analyzed studies, most used a distance close to 23mm.^5^ ^,^ ^11^ ^,^ ^13^ ^-^ ^20^ ^,^ ^22^ ^-^ ^31^

It is important to note that in the T-loop, due to its more sophisticated design, the M/F ratio is not constant with higher activations because the shape of the loop changes as the loop deactivates.^5^ For example, as the space closure increases, the force decreases about 30% and the momentum decreases about 18% in case of a translation movement. This means that when the T-loop deactivates, the M/F ratio tends to increase.^14^

**Figure 1:**
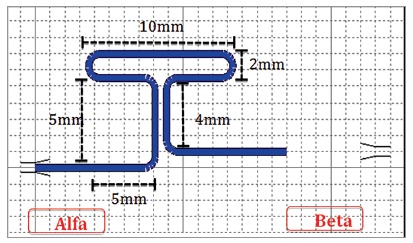
Illustration of the shape characteristics of the T-loop, made in the software Loop (dHAL Orthodontic Software, Athens, Greece), according to Kuhlberg and Burstone,^2^ 1997.

In summary, the higher the loop and the greater the amount of apical wire, the higher the M/F ratio obtained. For example, Figure 1 shows the dimensions of the T-loop proposed by Kuhlberg and Burstone^2^.

## T-LOOP PREACTIVATION

**Figure 2:**
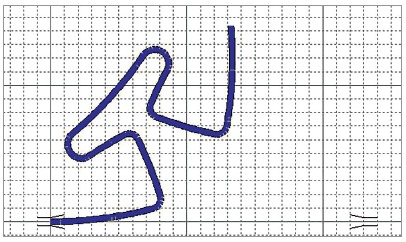
Illustration of the pre-activation of the T-loop, made in Loop software (dHAL Orthodontic Software, Athens, Greece), according to Kuhlberg and Burstone,^2^ 1997.

Because of anatomical limitations, it is not possible to sufficiently increase the loop to obtain the desired M/F ratio^5^. Thus, it is necessary to add larger moments to the loop, obtained bu means of preactivation (Fig 2). Several ways of preactivation have been suggested in the literature, such as preactivation by gable bends, curvature and concentrated bends.

**Figure 3:**
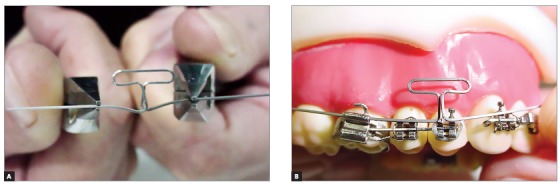
Example of neutral position evaluation. Note that the vertical arms of the loop intersect (A), which should be corrected for only a slight approach in the neutral position (B).

The main issue to be considered when adding preactivation is the neutral position. The neutral position is the position of the loop where only moments are used to insert the loop on the auxiliary tubes, i.e., there is no horizontal force; so when the loop is closed, the vertical legs practically abut,^5^ differently from what is shown in Figure 3, where the legs intersect. The moments in the neutral position are called residual moments.^9^ ^,^ ^12^ ^,^ ^15^ Several analyzed studies did not start from 0g when the loop was without opening of the vertical legs (0mm),^13^ ^,^ ^16^ ^,^ ^17^ setting up a methodological failure or bias when determining the neutral position. Other studies did not report force at 0mm of activation.^2^ ^,^ ^18^ The ideal, when adding preactivation, is to distribute the angular bends between the occlusal and apical portions of the loop, decreasing the possibility of the legs crossing.

When a gable bend is placed in the loop, i.e., an angle is positioned at the intersection between the horizontal and vertical legs, only in the occlusal region, the amount of activation is automatically increased as the legs intersect, and the neutral position is modified. If the orthodontist does not recognize this, he may be activating, for example, 2mm rather than 1mm. This can lead to permanent deformation in the loop and/or release of very low moments, leading to undesired uncontrolled movements.^6^

**Figure 4:**
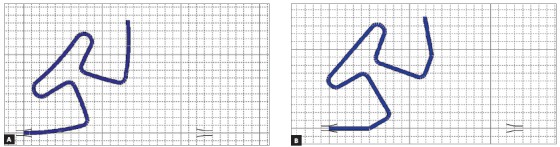
Images made in Loop software (dHAL Orthodontic Software, Athens, Greece), illustrating the types of pre-activation: A) pre-activation by curvature; B) preactivation by concentrated bends.

In addition to the gable, the T-loop can be preactivated by curvature or concentrated bends, as shown in Figure 4. The curvature bends promote a better internal distribution of stress during bends, since the bending moment is distributed throughout the thread. This reduces the chance of permanent deformation,^19^ making possible to form larger preactivations on the wire.

The concentrated bends are angled bends, but do not occur exactly between the horizontal and vertical legs of the loop. As the gable bends, they present a risk of permanent deformation due to stress relaxation,^17^ ^,^ ^20^ compromising the microstructure of the thread due to small breaks.^19^

Manharstberger et al^13^ (1989) found different values of force systems in different preactivations than Martins et al^20^ (2008) and Caldas et al^17^ (2011), although all agree that higher M/F ratios and lower magnitudes of force occur in the curvature group.^13^ ^,^ ^17^ ^,^ ^20^ It is more likely that the higher magnitudes of force occurred due to lack of adjustment of the neutral position in the study of Manharstberger et al.^13^ This means that the distance used between the vertical ends of the loop can lead to error, which makes difficult to compare the loops.

## T-LOOP ALLOYS AND ITS CHARACTERISTICS

Different alloys can be used for the construction of the T-loop. This changes the stiffness, the amount of activation and may increase or decrease the risk of plastic deformation, because it changes the maximum force and moment released by the loop.^9^ Several experimental studies were carried out in order to establish the correct force system of titanium-molybdenum alloys (also known as beta-titanium or TMA), stainless steel, and nickel-titanium (NiTi). In general, TMA releases 42% less force than stainless steel; thus, normally stainless steel alloys are not the first choice for the T-loop.^21^ This is compatible with the results of Maia et al,^22^ who compared, by means of a photoelastic analysis, two T-loops with the same conformation, but one of stainless steel and the other of titanium-molybdenum. The magnitude of force produced by the stainless steel loops was higher than those of TMA.^22^

The first T-loop was referenced in the literature in 1976, which was made of stainless steel, since the TMA alloy had not yet been developed. Its initial force system was characterized by an M/F ratio of approximately 6, in 7-mm activation.^5^ One way to increase the M/F ratio in stainless steel T-loops is heat treatment, as observed by Chen et al,^23^ in a T-loop pre-activated in gable at 30 degrees. However, even with heat treatment, the M/F ratio of the loops tested by the authors was between 5 and 6.8 mm,^23^ insufficient for translation.^7^

A trial activation should be performed to evaluate the stability of the T-loop, since its force system can change if the spring initial shape changes. It is done simulating several times the activation of the loop, outside the mouth, and then its format is evaluated in a template.^15^ In addition, some authors recommend overbending the loop to reduce the possibility of deformation, and then returning it to the desired position. If this is done, the activation in the mouth will be in the same direction as the last bend used (Bauschinger effect).^9^ A trial activation influences the force system of the because it reduces the risk of plastic deformation. However, only a few articles that experimentally evaluated the force system of TMA or stainless steel loops described their evaluation.^2^ ^,^ ^5^ ^,^ ^11^ ^,^ ^17^ ^,^ ^22^

Even performing the trial activations correctly, the plastic deformation also depends on the time. The deformation as a function of time, also defined as stress relaxation, depends on the intensity of stress and temperature, as high stresses and high temperatures favor the dislocation movements.^17^

When studying the stress relaxation in the preactivation by concentrated bends, they were found to have a progressive decrease of the load over time. This effect was critical in the first 24 hours, reducing the momentum, resulting in a approximate 1-mm decrease in the overlap of the vertical legs, causing a reduction of the force for a given activation.^17^ Preactivation in curvature better distributes stress and causes fewer failure in the wire, reducing deformation through stress relaxation. Larger magnitudes of force were found in the group with curvature, when adjusted to the neutral position, as well as higher M/F.^17^ Unlike stainless steel, NiTi has lower strength levels, mainly due to the superelastic plateau; however, it is not malleable, so specific devices are needed for conformation of the loop, which makes it difficult to use.^9^ Almeida et al^26^ found M/F ratios insufficient for translatory movement, but sufficient for controlled inclination on a 0.016 x 0.022-in NiTi wire. However, according to the authors, the released force was insufficient for en-masse retraction. The 0.017 x 0.025-in NiTi wires produced sufficient forces for mass retraction but did not reach M/F for controlled tipping^25^.

Rose et al^27^ compared 0.018 x 0.025-in TMA and NiTi loops. Both groups were preactivated at 0, 15 and 30 degrees. Activation was 7mm for TMA and 10mm for NiTi. The loops without preactivation failed to produce an optimal force system for translation. The NiTi loops at 30^o^ preactivation showed a higher M/F ratio than TMA at 30^o^ and lower magnitude of force, as well as lower load/deflection. The M/F ratio for the group with 30^o^ preactivation ranged from 10.1 in 5-mm activation to 39.9 in 0.5-mm activation, releasing forces between 50 and 150g, when reached an M/F ratio of 10.

Keng et al,^28^ in a split-mouth study, verified the same rate of space closure and angulation of the teeth using a TMA and a NiTi loop, but highlighted that NiTi has a higher elastic variation and a lower risk of fatigue.

## TYPES OF ANCHORAGE AND T-LOOP

Burstone^6^ dictated three types of anchoring needs: A) for cases where the posterior region needs to remain in position; B) where it is required a space closure of equal magnitude in the anterior and posterior regions; and C) where posterior protraction is necessary.^6^

### T-loops for type A anchorage

The key to control the posterior segment anchorage during the anterior retraction is not only the low magnitude of force, but the high M/F ratio in the posterior region, tending to a root movement and increasing as the loop deactivates. Anterior tooth movement is initially idealized by a controlled inclination, with the center of rotation positioned in the region of incisors apex, increasing the M/F ratio until a translation movement is obtained.^6^ Unfortunately, as the M/F ratio increases, the magnitude of force decreases. But translation and root movement require larger magnitudes of force than the inclination;^9^ therefore, devices for root correction are indicated after T-loop therapy for type A anchorage.^6^

**Figure 5:**
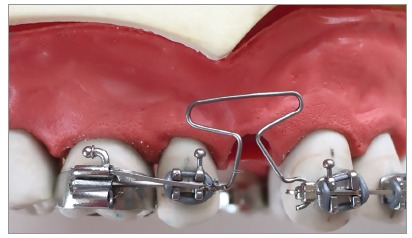
Composite T-loop according to Burstone,^6^ 1982.

Using a lower stiffness wire in the anterior region of the T-loop results in a smaller magnitude of force and a smaller magnitude of momentum, regardless the angulation method employed.^4^ ^,^ ^6^ Burstone^6^ idealized a composite TMA T-loop for anterior retraction (anterior region and loop: 0.018-in TMA; posterior region: 0.017 x 0.025-in TMA) (Fig 5), with a height of 7mm, apical length of 10mm, preactivated with an alpha (anterior) angulation at 105 degrees and a beta (posterior) angulation of 25 to 35 degrees. This loop produces an initial force of 200 grams in a 6-mm activation, with anterior M/F of 5.6 and posterior M/F of 12.8.^6^ However, although the force magnitude was favorable,^10^ a moment-to-force ratio of 5.6 still seems to be sufficient only for controlled inclination.^6^

**Figure 6:**
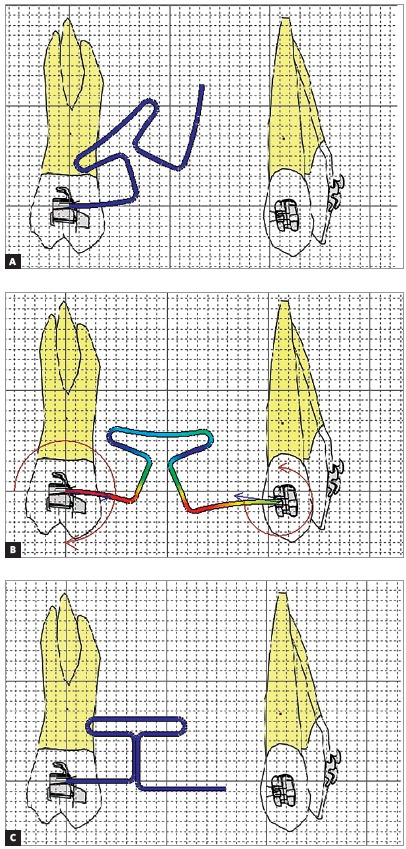
Illustration of the T-loop eccentrically positioned, according to Kulhberg and Burstone,^2^ 1997: A) preactivated loop inserted in the molar; B) loop with activation and C) neutral position of the loop.

There are other methods of obtaining differential moments in addition to angulation and cross-section modifications, such as working with the eccentric positioning of the loop. Even 1mm of eccentricity produced a noticeable difference in alpha (anterior) and beta (posterior) moments in the study of Kulhberg and Burstone^2^(Fig 6). The studied loop had 7 mm of height, 10 mm of apical length, and was conformed in 0.017 x 0.025-in TMA wire. When the loop moved 1, 2 or 3 mm to posterior, the anterior M/F ratio, at 6mm of activation, was between 3.7 and 2.22,^2^ insufficient for controlled tipping^3^, since with the increase of the eccentricity to posterior, the wire becomes very flexible in the anterior region, releasing a lower moment^12^. Although the 4-mm activation of this loop produced differential moments, the anterior M/F ratio was between 5.1 and 3.2. The posterior region had M/F ratio compatible with controlled inclination, which guarantees better control of the tooth apex.^3^ The authors also stated that the positioning may still be more critical with smaller interbrackets distances. The force increased with the increase of the eccentric positioning from 6 to 8mm. The horizontal force ranged between 340g in total activation and 0g, with no activation.^2^

**Figure 7:**
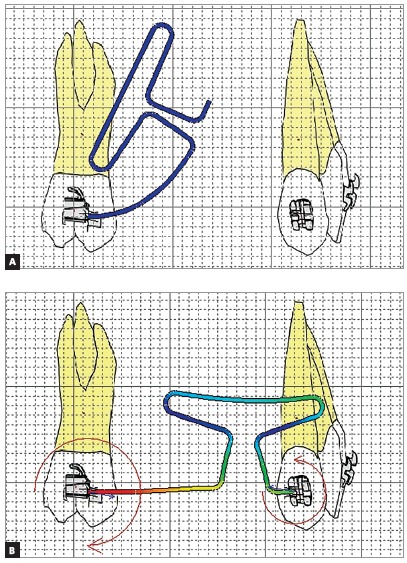
Images made in Loop software (dHAL Orthodontic Software, Athens, Greece), illustrating the types of pre-activation: A) self-correcting T-loop with pre-activation inserted into the molar tube; B) activated loop, according to Viecilli, ^16^ 2006.

Another way to achieve a large variation of activation in a 0.017 x 0.025-in TMA T-loop, with the spring off-centered to anterior, but still obtaining adequate differential moments for type A anchorage, is to add a preactivation only in the posterior region of the loop^12^ ^,^ ^16^(Fig 7). However, one important aspect that should be evaluated when making experimental measurements with the eccentric T-loop are the angles and steps formed by the different inclinations and vertical forces. When evaluating the steps and angles in a T-loop with an apical length of 16 mm, height of 8 mm, with the T off-centered to the anterior, and posterior preactivation, Viecilli^16^ obtained different force systems when considering the different steps and angles. The author did not consider the movement of the posterior unit and estimated a constant CRot (center of rotation) positioned at the height of the root apex. Such simplifications may be different from what occurs *in vivo*. The author's measurements are the ones closest to an initial controlled tipping movement in a T-loop with type A anchorage. In addition, the loop has good range of activation and does not require soldering, facilitating its clinical use.^16^

Some clinical studies have been conducted in relation to canine retraction with type A anchorage, in order to assess whether the stimulus-response force system is compatible with the data found in the experimental tests. The results were consistent with the prescribed force system, with a lower movement of the posterior anchorage unit than the anterior one.^24^ ^,^ ^29^ ^,^ ^30^ There was a difference in movement between the upper and lower canines, there was a controlled inclination of the upper canines, but not the lower one. According to the authors, this may be due to a greater distance from the line of action of the force and the CR (center of resistance) in the mandible. If the mandible offers greater resistance to movement than the maxilla, it dislocates the mandibular CR more apically, which explains the observed differences. Although the space closure was differential, the molars did not translate.^28^ The responses to the vertical forces were also different from the experimental studies. The limited tolerances that occur in physics experiments were not found. The vertical forces were different than expected, with no significant extrusion of the molar region.^24^ ^,^ ^29^ ^,^ ^30^ Even with different heights and preactivations, the results were similar.

### T-loops for type B anchorage

Patients requiring a space closure of equal intensity of the anterior and posterior regions may use type B anchorage mechanics. Translation of the two segments requires higher magnitudes of force, and the center of rotation is not constantly maintained in the two units, which can be verified through the experimentally obtained M/F ratios for several types of T-loops. The loop is positioned symmetrically so that it has practically the same M/F ratio in both brackets. 

**Figure 8:**
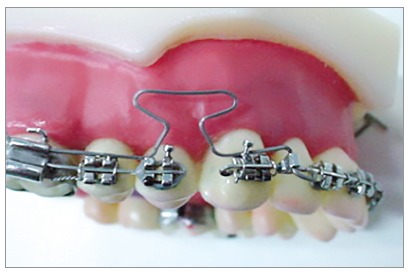
Images of the symmetrical T-loop, according to Burstone,^6^ 1982.

Burstone^6^ developed a symmetrical T-loop with 7 mm in height, 10 mm in apical length, and with preactivation by curvature, according to a template (Fig 8). In a 7-mm activation, the initial M/F ratio in both units suggests a controlled inclination movement, approaching a translation, in average, in 3 mm of activation of the loop, considering for translation an M/F near 10. Due to the low magnitude of force in 4 mm of loop activation, it is recommended to reactivate the loop when reaching this space closure.^9^ It was observed that, in a 0-mm activation, 0g of horizontal force was obtained, which determines a correct evaluation of the neutral position of the loop^6^. It is also possible to open the ears of the loop and apply a curvature in the region of the legs of about 40 degrees.^31^ Kuhlberg and Burstone^2^ analyzed the symmetrical loop with the same configurations as Burstone.^6^ The loop presented higher magnitudes of forces and lower M/F ratios. This may have occurred because of a change in the neutral position in the study by Kulhberg and Burstone^2^.

**Figure 9:**
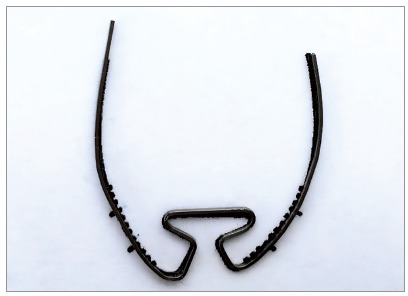
Template for T-loop conformation, according to Hoenigl et al,^18^ 1995.

Hoenigl et al^18^ evaluated a higher loop, with 8.5 mm in height, 10 mm in apical length and 7 mm in total activation (Fig. 9). The magnitude of force with a greater height decreased (200g), and the moment/force ratio increased (between 7.6 and 9.2),^18^ when compared with other loops with the same characteristics, but with lower heights.^2^ ^,^ ^13^ ^,^ ^17^ ^,^ ^20^ However, after 3mm of loop deactivation, the magnitude of force is below ideal for a translation movement,^10^ which generates the need for reactivation.

Manharstberger et al,^13^ Martins et al^20^ and Caldas et al^17^ analyzed similar loops, where the first differs from the second and third only by 1mm in height. The authors studied the different preactivations for two cross-sections: TMA 0.017 x 0.025-in and 0.016 x 0.022-in. Comparing a 0,016 x 0,022-in T-loop with only 5mm of activation and a 0.017 x 0.025-in T-loop with 7-mm activation, 47% less force is produced and M/F is 23% higher.^13^ Working with a different wire stiffness changes the M/F ratio, since it generates smaller magnitudes of force and momentum.^13^ However, there was a disparity in the force magnitude values ​​of these three studies. Manharstberger et al^13^ found values ​​close to 350g in the 0.017 x 0.025-in loops. Martins et al^20^ found values ​​between 456 and 516g, in 5mm of activation (starting from -2mm, by crossing the legs, up to 0mm of activation of the loop).^20^ Caldas et al^17^ found values ​​between 404.7 and 431.5g. The magnitudes of force obtained in both groups seem very high for controlled tipping.^10^

### T-loops for type C anchorage

Posterior space closure is challenging, because anterior teeth have less support to provide anchorage.^9^ The loops for type C anchorage follow the same principle of differential moments as the type A anchorage, but the logic and the force is reversed.^6^

Burstone^6^ reported in his paper two types of loop for posterior protraction. The first one was conformed in 0.017 x 0.025-in TMA, with a height of 7 mm and 10 mm of apical length, positioned posteriorly (1/3 of the interbrackets distance from the molar tube) and had larger angulation bends in alpha. A 4-mm activation was recommended. The initial released force system was 309g, M/F of 8 in alpha and 4.4 in beta. Vertical forces were present and had a magnitude of 40.3g. The loop was positioned decentralized to posterior, which produces a more constant CRot in the beta region. In the alpha region, if these teeth move, they tend to move forward rather than backward.^6^ Kuhlberg et al^2^ evaluated the effect of the off-centering of the loop with the same parametric characteristics as the symmetrical loop of Burstone^6^, but shifted 1, 2 and 3 mm to the anterior. The anterior M/F ratios at 4-mm activation varied between 9.2 and 10.5 in alpha and between 6.6 and 4.9 in beta.^2^

When the vertical forces acting on the first T-loop described by Burstone^6^ are not indicated for the patient, intermaxillary elastics associated with the symmetrical loop activated in 4 mm can be used. A differential M/F ratio is produced by the different forces produced in the anterior and posterior units. However, it is worth noticing that the use of intermaxillary elastics can alter the occlusion plane, especially Class II elastics, by extruding the incisors in a Class II patient. When analyzing the force system of two magnitudes of elastic force, the author did not consider the acting vertical forces, only the horizontal forces. At 4-mm activation, the 150g elastic generates an M/F ratio of 10.9 in alpha and of 6 in beta, with a posterior force of 335g. By adding a 100g elastic, the same M/F ratio is obtained, however the posterior force magnitude drops to 285g.^6^

## CANINE RETRACTION T-LOOP

For this type of loop, the first important issue to be addressed is whether the retraction will be done with all the six anterior teeth or canine separate. The magnitudes used for en-masse retraction are practically the same as the magnitudes used for retraction only of the canine, since low magnitudes of force retract all the six teeth^6^. So, there is no difference in the loss of molar anchorage between the two approaches. However, it is not recommended to retract first the canine and then the incisors, except when spaces are needed for anterior alignment, since the retraction in two stages, besides being aesthetic, increases treatment time.^9^

**Figure 10:**
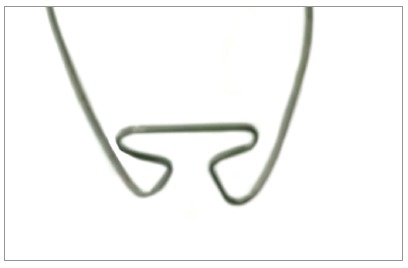
Illustration of the anti-rotation bends aiming to determine rotational moments of the T-loop for canine retraction, according to Burstone,^6^ 1982.

Due to the distance between the center of resistance and the loop in the occlusal view, anti-rotation bends are required so that the canine does not rotate its distal while retract^9^, as shown in Figure 10. The force system is identical to that of the other loops, the only change is the anti-rotation bends. Therefore, the canines can be activated for type A, B and C anchorage.

## FINAL CONSIDERATIONS

The space closure T-loops are frequently used in orthodontic mechanics, and several formats are reported in the literature. Studies indicate that the higher the loop and the greater the apical length, the higher the M/F ratio and the lower the released force. However, even with these parametric characteristics, the preactivations are fundamental to obtain adequate M/F ratio, in order to produce controlled inclination or translation.

Most of the studies did not evaluate the neutral position, which makes complex the comparison of force systems of the loops. In addition, some types of preactivation influence plastic deformation, such as those with concentrated angles. The trial activation is fundamental so that there is no permanent deformation in the shape of the loop, being essential for comparing force systems. Thus, it is indicated to check the shape of the loop at each patient return, to verify if there was any change, in order to obtain the planned movement in all phases of spaces closure.

A constancy of the force would be ideal, being obtained with the superelastic plateau of the NiTi wires. These wires need to be better evaluated because of their great potential of use, since the studies, until now, have found very small force levels.

Several ways of obtaining differential moments have been suggested in the literature. Some studies do not report the vertical movement of the units that hold the loop in position during the tests, and the steps and angles that occur are neglected. However, clinical studies seem to be less rigid in relation to the results of vertical forces, since chewing itself can compensate for these forces.

For the symmetrical loop, there was no consensus on the horizontal forces, and there was a great deal of discrepancy between the studies. Most of the loops can release initial M/F compatible with controlled inclination. The main point is that, as the loop deactivates and the M/F ratio increases, the force decreases; however, the magnitudes of force required for translation and root movement are greater.

The loops for posterior protraction were the least studied and seem to be the most challenging. The eccentric positioning in these loops seems to have a better influence than in the type A loop. The use of intermaxillary elastics is well indicated; however, has the risk of compromising the occlusal plane, being more critical with the use of elastics in Class II patients. The great potential for future studies with this type of loop is noticeable, taking into account that, if properly activated, they provide favorable force systems to obtain a differential space closure. There is, however, a need to standardize the methodology to conform the loops, taking into account the variables that influence the force system, in order to perform more accurate tests, obtain more accurate force systems and compare studies more effectively.
